# The *miRNA-29b* Is Downregulated in Placenta During Gestational Diabetes Mellitus and May Alter Placenta Development by Regulating Trophoblast Migration and Invasion Through a *HIF3A*-Dependent Mechanism

**DOI:** 10.3389/fendo.2020.00169

**Published:** 2020-03-31

**Authors:** Da-Guang Sun, Shi Tian, Lu Zhang, Yi Hu, Chun-Yi Guan, Xu Ma, Hong-Fei Xia

**Affiliations:** ^1^Reproductive and Genetic Center of National Research Institute for Family Planning, Beijing, China; ^2^Graduate School, Peking Union Medical College, Beijing, China; ^3^Chongqing Key Laboratory of Birth Defects and Reproductive Health, Chongqing, China; ^4^Maternal and Child Health Hospital, Beijing, China

**Keywords:** *miR-29b*, gestational diabetes mellitus, *HIF3A*, placenta, human

## Abstract

Gestational diabetes mellitus (GDM) is a disease that changes the function of microvascular of placenta. MicroRNA (miRNA) expression in placenta may contribute to the pathogenesis of GDM. Here, we evaluate the role and function of *miR-29b* in the development of GDM. This study discovered that *miR-29b* expression was lower in placentas derived from patients with GDM than that in control placentas. *MiR-29b* over-expression inhibited cell growth and migration, and *miR-29b* knockdown promoted cell migration. Then we predicted and confirmed that *HIF3A* was a direct target of *miR-29b* with two specific binding sites at the recognition sequences of *miR-29b* in 3′-UTR of *HIF3A* mRNA, which was negatively correlated with *miR-29b* expression level. The up-regulation of *HIF3A* partially antagonized the inhibitory effect of *miR-29b* over-expression on cell growth and migration. The enhancement of cell migration induced by *miR-29b* knockdown was attenuated by down-regulating *HIF3A*. These results imply that down-regulation of *miR-29b* may be related with the development of GDM partially via increasing the expression of *HIF3A*, which may provide a new insight for the mechanism of GDM.

## Introduction

Gestational diabetes mellitus (GDM), defined as any degree of glucose intolerance with onset or first recognition during pregnancy, is one of the most common complications during pregnancy (1). Its prevalence varies worldwide by applying the characteristics of the underlying population and the diagnostic criteria. The highest prevalence over the past decade was reported in Middle East and North Africa, with a median estimate of 13%, whereas the lowest was in Europe, with a median prevalence of 5.8% (2, 3). Women with GDM have an increased risk of maternal and fetal complications during pregnancy as well as long-term adverse health outcomes for both mother and offspring (4). GDM has a variety of pathogenic factors, and may be the result of co-effect between genetic and environmental factors. However, the precise pathogenesis of GDM still needs further research.

MicroRNAs (miRNAs) are a class of single-stranded small-molecule non-coding RNAs with a length of 18–23 nucleotides and highly conserved in the evolutionary process that play a negative regulatory role at the epigenetic level. Recent studies have found that more and more miRNAs play an important role in the pathogenesis of GDM (5–7). Li et al.'s study shows that miR-96 is down-regulated in GDM samples and protects pancreatic β-cell function by targeting PAK1 in GDM (8). High glucose suppresses the viability and proliferation of HTR-8/SVneo cells through regulation of the miR-137/PRKAA1/IL-6 axis (9). Up-regulation of *miR-98* in the placental tissues of human GDM is linked to the global DNA methylation via targeting MECP2 (10). In our previous study, we used miRNAs chip to screen for differentially expressed miRNAs in placenta between GDM and normal control in order to explore the molecular biological regulation mechanism of miRNAs in GDM. *MiR-29b* was differentially expressed in placenta, but the functions and molecular mechanism of *miR-29b* in GDM were still unclear.

In this study, we validate the expression patterns of *miR-29b* in a large cohort of GDM patients and study the roles of *miR-29b* using the trophoblast cell line. Meanwhile, we analyze the potential molecular mechanisms of *miR-29b* in GDM.

## Results

### Down-Regulation of *miR-29b* Expression in the Placental Tissues From Patients With GDM

**Figure 1:**
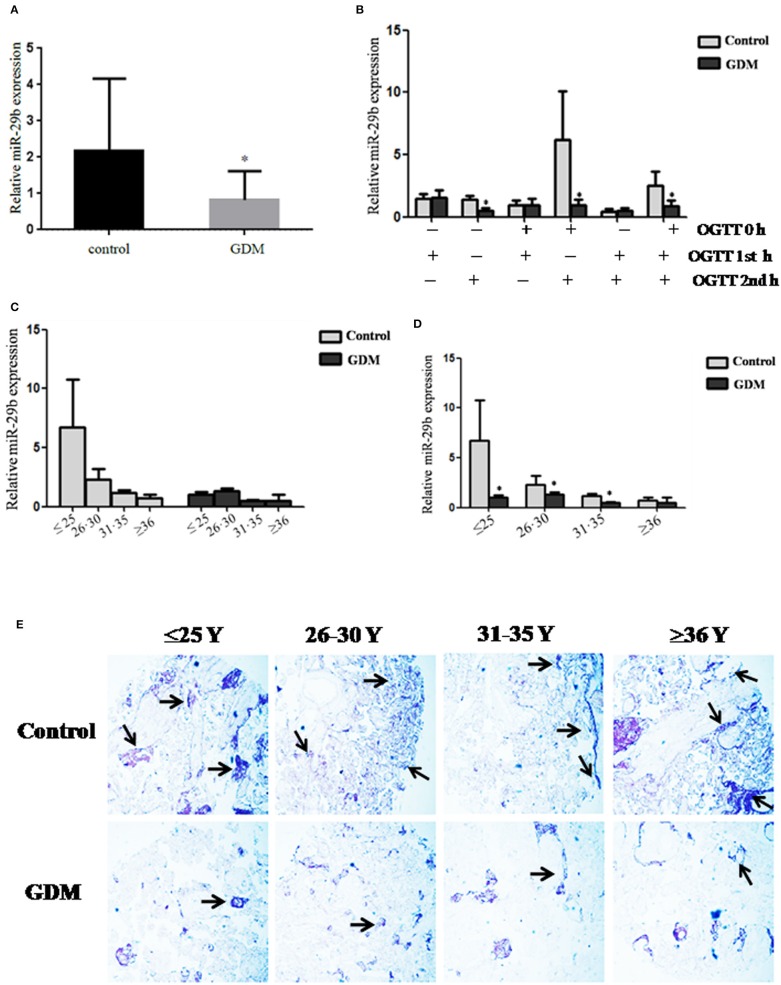
Down-regulation of *miR-29b* in the placental tissues from patients with GDM detected by qRT-PCR. The overall expression trend of *miR-29b* in all placental tissues of GDM group was lower than that in control group. **(A)** The GDM patients were grouped into six groups according to OGTT values in blood, including 1st h, 2nd h, 0 and 1st h, 0 and 2nd h, 1st and 2nd h, 0 and 1st and 2nd h **(B)**. The GDM placental tissues were also grouped into four groups according to age, including ≤25 Y, 26–30 Y, 31–35 Y, and ≥36 Y. There was a trend toward a decrease in the level of expression *miR-29b* in normal placenta with age **(C)**. *MiR-29b* expression level was significantly down-regulated in the groups of ≤25 Y, 26–30 Y, and 31–35 Y as compared with matched control **(D)**. Random 10 pair samples (*n* = 10) in each age group (OGTT 1st h, 2nd h, 0 and 1st h, 0 and 2nd h, 1st and 2nd h, 0 and 1st and 2nd h); **(B)** and random 15 pair samples (*n* = 15) in each age group (≤ 25 Y, 26–30 Y, 31–35 Y, ≥36 Y); **(C)** were selected for qRT-PCR. Each sample in each experiment was detected in triplicate. *U6* serves as an internal reference to normalize the experimental error **(E)**. Placentas from 204 GDM patients and 202 normal pregnant women were separately arranged into two tissue microarrays according to the match between the case group and the control group, and detected by *in situ* hybridization. The stain was developed with BCIP/NBT. Black arrows indicate hybridization signals and the positive signals of *miR-29b* are blue. *P < 0.05.

The expression pattern of *miR-29b* in the placental tissues from patients with GDM and normal controls was determined by quantitative reverse transcription polymerase chain reaction (qRT-PCR). The overall expression trend of *miR-29b* in all placental tissues of GDM group was lower than that in control group (*P* < 0.05; Figure 1A). The GDM patients were grouped into six groups according to the abnormal oral glucose tolerance test (OGTT) values in blood, including 1st hour (h), 2nd h, 0 and 1st h, 0 and 2nd h, 1st and 2nd h, 0 and 1st and 2nd h. The expression of *miR-29b* was significantly reduced in the groups of 2nd h (*P* < 0.05), 0 and 2nd h (*P* < 0.05), 0 and 1st and 2nd h (*P* < 0.05; Figure 1B). Meanwhile, the GDM placental tissues were grouped into four groups according to age, including ≤ 25 years (≤ 25 Y), 26–30 years (26–30 Y), 31–35 years (31–35 Y), and equal to or over 36 years (≥ 36 Y). There was a trend toward a decrease in the level of expression *miR-29b* in normal placenta with age (Figure 1C). *MiR-29b* expression level was significantly down-regulated in the groups of ≤25 Y (*P* < 0.05), 26–30 Y (*P* < 0.05), and 31–35 Y (*P* < 0.05; Figure 1D) as compared with matched controls. The distribution of *miR-29b* in placenta was detected by *in situ* hybridization (Figure 1E). The positive staining of *miR-29b* was found in the surface of the syncytiotrophoblast membrane and villous stroma in both GDM patients and matched controls.

### *miR-29b* Over-Expression Inhibits Trophoblast Cells Growth

In order to detect the transfection efficiency of *miR-29b* mimic and inhibitor, *miR-29b* expression level in HTR8-/SVneo cells transfected with *miR-29b* mimic, miRNA mimic negative control, *miR-29b* inhibitor, or miRNA inhibitor negative control was detected by qRT-PCR. *MiR-29b* mimic significantly enhanced the expression level of mature *miR-29b* (*P* < 0.01; Figure S1A). Mature *miR-29b* expression was evidently reduced by *miR-29b* inhibitor (*P* < 0.01; Figure S1B).

**Figure 2:**
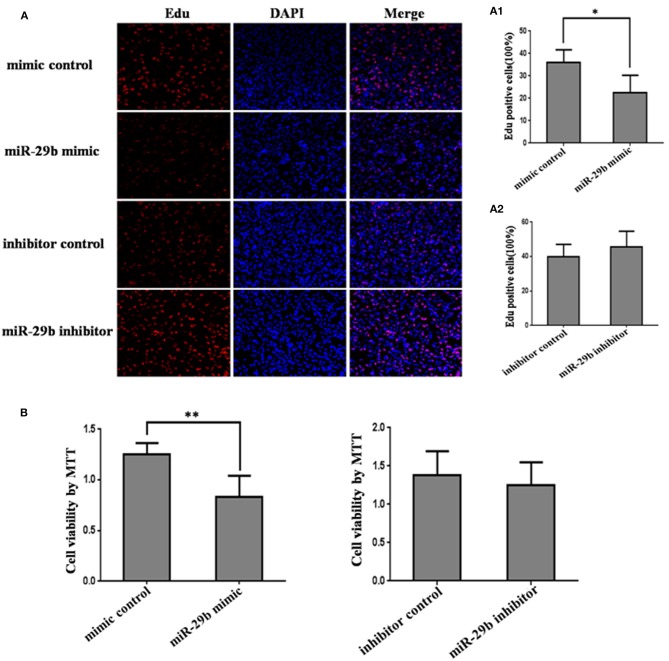
The effect of *miR-29b* on trophoblastic cells proliferation. HTR8 cells were transfected with the miRNA mimic control, *miR-29b* mimic, miRNA inhibitor control or *miR-29b* inhibitor, respectively. After 48 h of transfection, cell proliferation was determined by EdU assay **(A, A1, A2)** and MTT **(B)**. Each treatment group had three replicates and the experiment was repeated for three times (*n* = 9). *P < 0.05, **P < 0.01.

The effects of *miR-29b* on cell growth were analyzed by MTT assay and EdU assay (Figure 2). The results showed that *miR-29b* over-expression inhibited cell viability and proliferation as compared with control group (*P* < 0.05; Figures 1B, 2A). There was no significant difference in cell viability and proliferation between *miR-29b* inhibitor and miRNA inhibitor negative control (Figures 2A,B).

### *miR-29b* Over-expression Promotes Late Apoptosis of Trophoblast Cells

**Figure 3:**
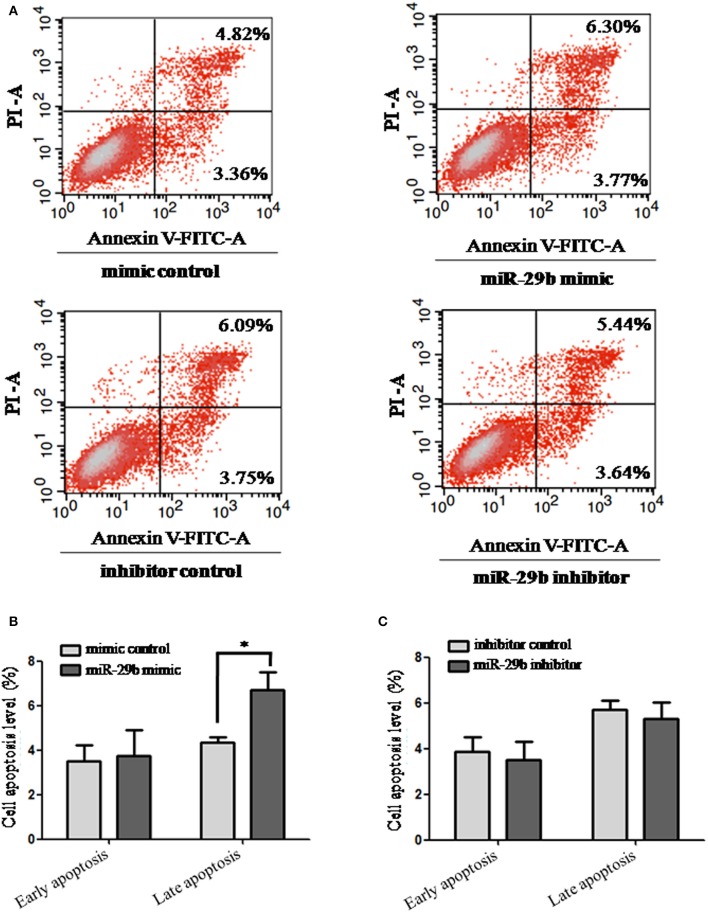
Apoptosis in trophoblastic cells was detected by flow cytometry. HTR8 cells were transfected with the miRNA mimic control, *miR-29b* mimic, miRNA inhibitor control or *miR-29b* inhibitor, respectively, for 48 h. Then single cell suspension was prepared and stained with annexin V/PI and subjected to flow cytometry analysis **(A)**. Lower left quadrant, viable cells (annexin V-FITC and PI negative); lower right quadrant, early apoptotic cells (annexin V-FITC positive and PI negative); upper right quadrant, late apoptosis/necrosis cells (annexin V-FITC and PI positive). The percentage of early and late apoptotic cells (representatives of three separate experiments) is shown in the lower right and upper right panels, respectively. The histograms, respectively, represent the average percentage of apoptosis cells in cells treated by *miR-29b* mimics **(B)** and *miR-29b* inhibitor **(C)**. The experiment was repeated for three times (*n* = 3). *P < 0.05.

The effects of *miR-29b* on cell apoptosis were detected by flow cytometry analysis (Figure 3). When trophoblast cells were transfected with *miR-29b* mimic, the percentage of apoptotic cells was significantly increased at late stage (*P* < 0.05; Figure 3B) and no statistical difference at early stage as compared with miRNA mimic negative control. No significant difference was observed in cell apoptosis level between *miR-29b* inhibitor and miRNA inhibitor negative control at both early and late stages (Figure 3C).

### *miR-29b* Regulates Trophoblast Cells Migration and Invasion

**Figure 4:**
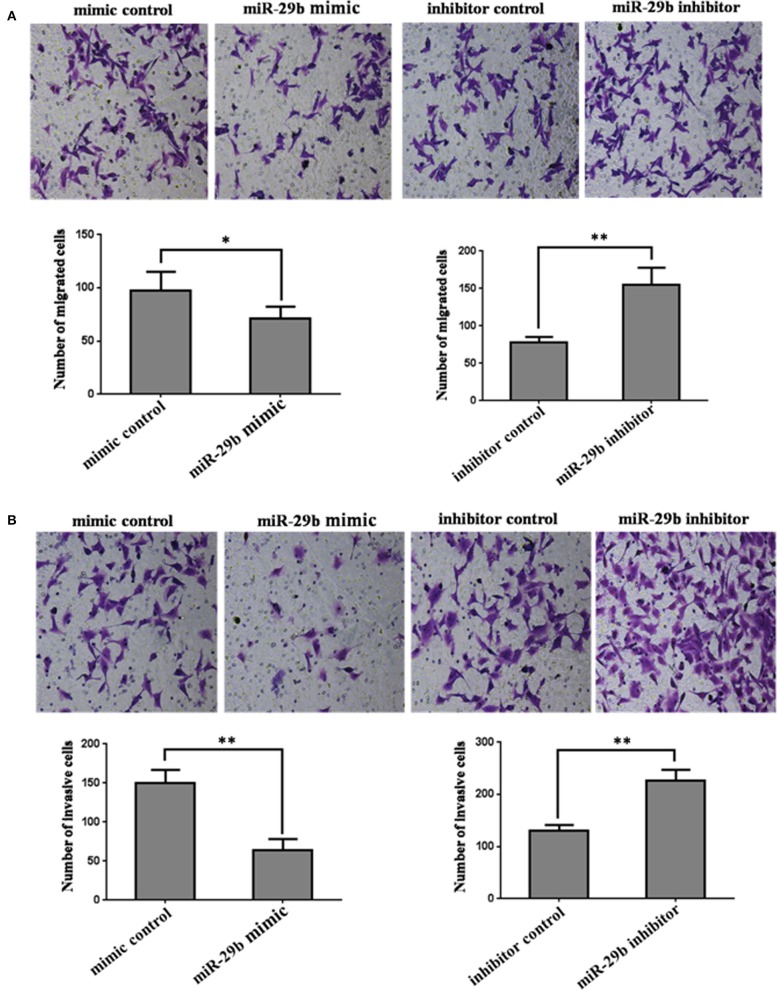
The effects of *miR-29b* on cell migration and invasion of trophoblastic cells. HTR8 cells were transfected with the miRNA mimic control, *miR-29b* mimic, miRNA inhibitor control or *miR-29b* inhibitor, respectively. Cells were harvested 48 h after transfection and recounted to 0.5 × 10^6^ cells/ml in every group to seed Transwells for cell migration assay **(A)** or matrigel-coated Transwells for cell invasion assay **(B)**. At time of harvest, the cells on top of the membranes were removed, and the cells on the bottoms of the membranes were stained with haematoxylin and eosin. The cell migration or invasion was quantified by counting the amount of cells passing through the membrane from five different fields per sample at 100 × selected in a random manner after 17 h or 24 h of incubation. Histogram represents the number of migrated or invaded HTR8 cells. Data are expressed as the mean numbers of independent triplicate experiments. The experiment was repeated for three times (*n* = 3). *P < 0.05, **P < 0.01.

In order to further analyze the role of *miR-29b* in the occurrence of GDM, the effects of *miR-29b* on trophoblast cell migration and invasion were evaluated by a Transwell chamber. The number of migrating cells was significantly decreased in *miR-29b* mimic group compared with mimic control (*P* < 0.05) and markedly increased in *miR-29b* inhibitor group compared with inhibitor control (*P* < 0.01; Figure 4A). The number of cell invasion was less in *miR-29b* mimic group than that in mimic control group (*P* < 0.01) and more in *miR-29b* inhibitor group than that in inhibitor control group (*P* < 0.01; Figure 4B).

### Screening and Identifying of Target Gene of *miR-29b*

**Figure 5:**
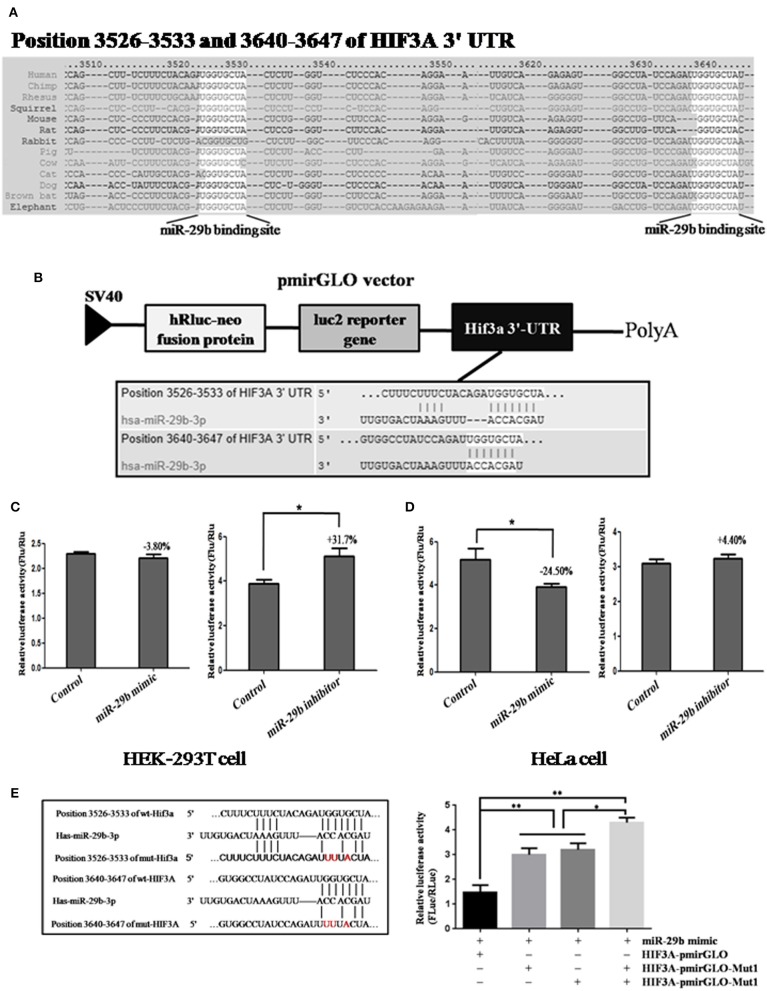
The prediction and confirmation of the *miR-101* target *HIF3A*. **(A)** *MiR-29b* binding sites in the 3′-UTR region of *HIF3A* in cross-species. **(B)** Schematic diagram for constructing the *miR-29b* binding site into pmirGLO vector. **(C,D)** Confirmation of the target gene of *miR-29b*. For dual-luciferase assay, HEK-293T cells and HeLa cells were co-transfected with miRNA mimic control, *miR-29b* mimic and *HIF3A*-pmirGLO; HeLa cells were co-transfected with miRNA inhibitor control or *miR-29b* inhibitor and *HIF3A*-pmirGLO. **(E)** Mutation analysis of the *miR-29b* binding sites. When the binding sites of *miR-29b* in the 3′-UTR of *HIF3A* was mutated (*HIF3A*-pmirGLO-Mutant 1 or 2). Each treatment group was repeated for *three* times for independent triplicate experiments (*n* = 9). The results were expressed as relative luciferase activity (Firefly LUC/Renilla LUC). ^*^P < 0.05, ^**^P < 0.01.

To discover the possible molecular mechanisms through which *miR-29b* may play roles in cell growth and migration, the target genes of *miR-29b* were predicted online by Targetscan, PicTar and miRanda and a large number of putative miRNA targets were found. We focused on genes associated with cell proliferation and apoptosis. Inhibitor of growth protein-2 (ING2), inhibitor of growth protein-3 (ING3) and hypoxia-inducible factors 3A (*HIF3A*) were predicted to be the target genes of *miR-29b*. The 3′-UTR segment of human ING2, ING3, or *HIF3A* among different species was conservative (Figures S2A, S3A and Figure 5A).

In order to confirm whether these genes were the target genes of *miR-29b*, the 3′-UTR segment of human ING2, ING3, or *HIF3A* were cloned into pmirGLO vector, respectively (called ING2-pmirGLO, ING3-pmirGLO, *HIF3A*-pmirGLO; Figures S2B, S3B, and Figure 5B). ING2-pmirGLO, ING3-pmirGLO or *HIF3A*-pmirGLO was, respectively, co-transfected with *miR-29b* mimic or inhibitor to detect the luciferase activity in HeLa cells and HEK-293T cells (Figures S2C, S3C,D and Figures 5C,D). The stronger the luciferase activity, the stronger the expression of the target gene, that is, the weaker the binding of the *miR-29b* to 3′-UTR of the target gene. When cells were co-transfected with *miR-29b* mimic or inhibitor and ING2-pmirGLO or ING3-pmirGLO, the luciferase activity was no significant difference compared with control group. When cells were co-transfected with *HIF3A*-pmirGLO and *miR-29b* mimic or mimic control, the luciferase activity was weaker in *miR-29b* mimic group than that in mimic control group in HeLa cells (*P* < 0.05), but no significant difference in HEK-293T cells. When cells were co-transfected with *HIF3A*-pmirGLO and *miR-29b* inhibitor or inhibitor control, the luciferase activity was stronger in *miR-29b* inhibitor group than that in inhibitor control group in HEK-293T cells (*P* < 0.05), but no significant difference in HeLa cells. These results suggest that *miR-29b* may bind to 3′-UTR of *HIF3A*.

To further confirm whether *miR-29b* may bind to 3′-UTR of *HIF3A*, the potential recognition sequences of *miR-29b* in 3′-UTR of HIF3α mRNA was mutated (named as *HIF3A*-pmirGLO-Mut; Figure 5E). There were two potential *miR-29b* recognition sites within 3′-UTR of *HIF3A* mRNA. Compared with HIF3α-pmirGLO-Mut1 or/and *HIF3A*-pmirGLO-Mut2, the enzyme activity was significantly reduced in HeLa cells co-transfected with *miR-29b* mimic and *HIF3A*-pmirGLO (*P* < 0.01). Moreover, the luciferase activity was higher in both *HIF3A*-pmirGLO-Mut1 and *HIF3A*-pmirGLO-Mut2 group than that in *HIF3A*-pmirGLO-Mut1 or *HIF3A*-pmirGLO-Mut2 alone (*P* < 0.05). These data indicate that *miR-29b* can bind to the recognition element in 3′-UTR of *HIF3A*, and that *HIF3A* is a target gene of *miR-29b*.

### HIF3α Protein Level Is Inversely Regulated by *miR-29b in vitro*

**Figure 6:**
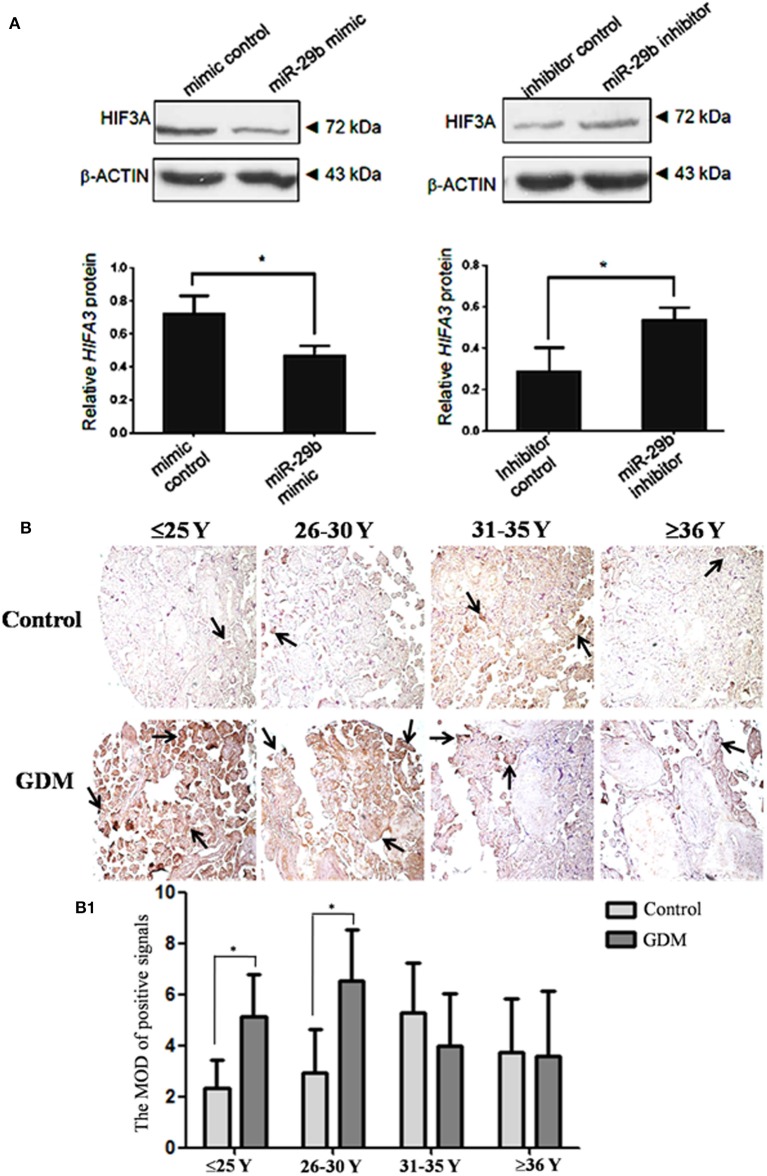
HIF3α protein level in the trophoblastic cells and placental tissues from patients with GDM. The expression of HIF3α in HTR8 cells was detected by western blotting **(A)**. The bands were analyzed using the Quantity One analyzing system (Bio-Rad Laboratory inc.). The expression of β-ACTIN served as an internal control. The black histogram represents the optical densities of the signals quantified by densitometric analysis and represented as HIF3α intensity /β-ACTIN intensity to normalize for gel loading and transfer (*n* = 3). The expression of HIF3α in the placental tissues from patients with GDM was detected by immunohistochemistry using anti- HIF3α antibody **(B)**. Placentas from 204 GDM patients and 202 normal pregnant women were separately arranged into two tissue microarrays according to the match between the case group and the control group. The tissue microarrays were incubated with normal goat serum as negative control. The stain was developed with DAB and the nuclei were stained with haematoxylin. The histograms, respectively, represent the MOD of positive signals **(B1)**. The MOD of positive signals was determined through Image J software with H-score grading system in three different optical fields selected in a random manner for each sample. Random 15 pair samples (*n* = 15) in each age group (≤ 25 Y, 26–30 Y, 31–35 Y, ≥36 Y) were selected for optical density analysis. Black arrows indicate positive immunoreaction. *P < 0.05.

Though *miR-29b* could bind to the recognition sequences in 3′-UTR of HIF3α, it was unclear whether *miR-29b* could regulate HIF3α expression in trophoblast cells. Here, we found that the protein level of HIF3α was significantly reduced in *miR-29b* mimic group as compared with mimic control group (*P* < 0.05). *MiR-29b* inhibitor significantly enhanced the protein level of HIF3α as compared with inhibitor control (*P* < 0.05; Figure 6A). These results show that *miR-29b* can inversely regulate HIF3α protein level.

### The Expression of HIF3α in GDM Placental Tissues

The HIF3α expression in the placental tissues from patients with GDM was detected by immunohistochemistry (Figure 6B). The positive signals of *miR-29b* were located in the apical membrane of the syncytiotrophoblast and villous stroma in different age groups in both the GDM patients and corresponding controls. The mean optical densities (MOD) of positive signals was determined through Image J software with H-score grading system in three different optical fields selected in a random manner for each sample. The MOD of positive signals was stronger in GDM group than that in matched controls in the groups of ≤25 Y and 26–30 Y (*P* < 0.05; Figure 6B1).

### *HIF3A* Is the Functional Target of *miR-29b*

**Figure 7:**
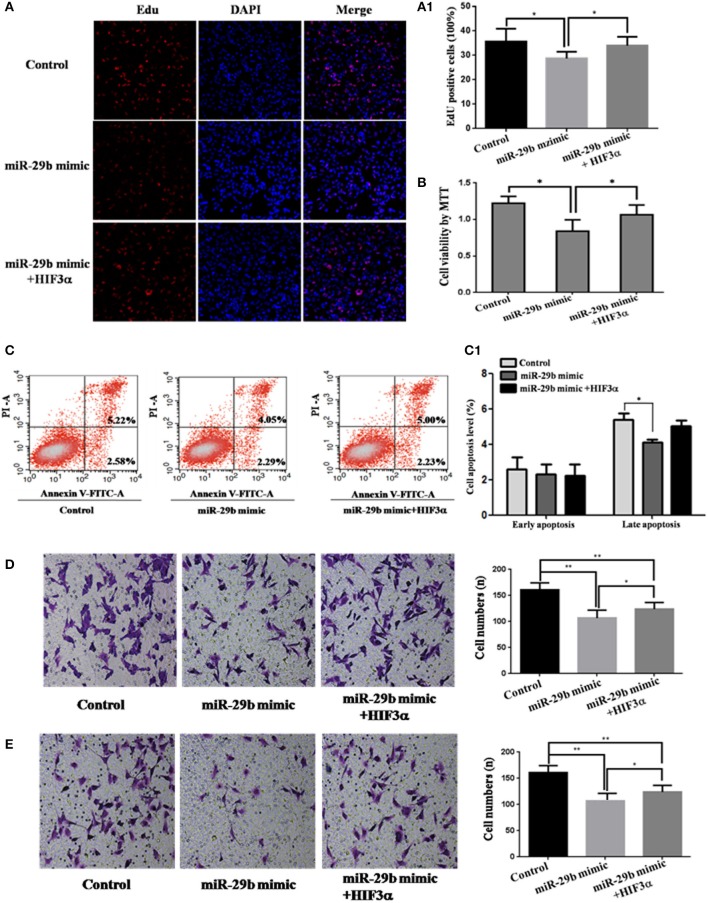
Over-expression of *HIF3A* partially restored the inhibition of cell growth induced by up-regulation of *miR-29b*. MiRNA mimic control and pcDNA3.1 control vector were co-transfected into HTR8 cells to serve as control. pcDNA3.1 control vector and *HIF3A*-pcDNA3.1 were severally co-transfected with *miR-29b* mimic. Cell proliferation was determined by EdU assay **(A, A1)** and MTT **(B)**. Each treatment group had three replicates and the experiment was repeated for three times (*n* = 9). Cell apoptosis were detected by flow cytometry **(C)** and the histogram represents the average percentage of apoptosis cells **(C1)**. The experiment was repeated for three times (*n* = 3). Cells were seeded Transwells for cell migration assay **(D)** or matrigel-coated Transwells for cell invasion assay **(E)**. The experiment was repeated for three times (*n* = 3). *P < 0.05, **P < 0.01.

**Figure 8:**
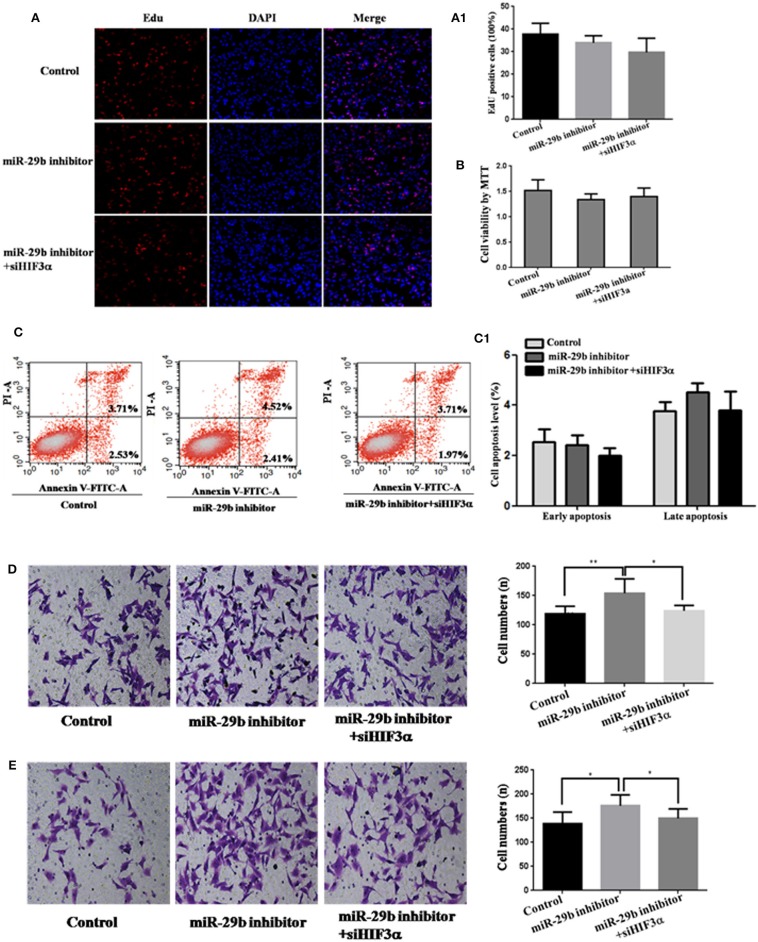
Knockdown of *HIF3A* attenuates the down-regulation of *miR-29b*-mediated enhancement of cell growth. MiRNA inhibitor control and scramble siRNA control were co-transfected into HTR8 cells to serve as control. Scramble siRNA control and *HIF3A* siRNA (*siHIF3A*) were severally co-transfected with *miR-29b* inhibitor. Cell proliferation was determined by EdU assay **(A, A1)** and MTT **(B)**. Each treatment group had three replicates and the experiment was repeated for three times (*n* = 9). Cell apoptosis were detected by flow cytometry **(C)** and the histogram represents the average percentage of apoptosis cells **(C1)**. The experiment was repeated for three times (*n* = 3). Cells were seeded Transwells for cell migration assay **(D)** or matrigel-coated Transwells for cell invasion assay **(E)**. The experiment was repeated for three times (*n* = 3). *P < 0.05, **P < 0.01.

To analyze whether *HIF3A* was the functional target of *miR-29b*, we performed the target gene compensation experiment (Figures 7, 8). *MiR-29b* mimic could inhibit cell proliferation (*P* < 0.05), migration and invasion. When *HIF3A* was over-expressed in cells transfected with *miR-29b* mimic, cell proliferation, late apoptosis, migration and invasion ability were stronger than that in cell transfected with *miR-29b* mimic alone (*P* < 0.05), close to control (Figure 7). *MiR-29b* inhibitor could promote migration and invasion. When *HIF3A* was knocked down in cells transfected with *miR-29b* inhibitor, migration and invasion ability were weaker than that in cell transfected with *miR-29b* inhibitor alone (*P* < 0.05), close to normal control (Figure 8).

## Discussion

Here, we found that the expression of *miR-29b* in placental tissues from GDM patients was observably reduced compared with that in normal controls. The positive signals of *miR-29b* were detected in the surface of the syncytiotrophoblast membrane and villous stroma. In rats with GDM, *miR-29b* expression was also diminished (11). These data suggest that the decrease of *miR-29b* may be correlated with GDM.

Now, it is generally believed that placenta plays a key role in the pathogenesis of GDM. The placenta is the only interface that connects the mother to the fetus. It is not only an important organ that participates in the exchange and circulation of substances between the mother and the fetus, but also has important endocrine functions (12, 13). After pregnancy, placenta synthesized placental prolactin, sex hormone and maternal adrenocortical hormone, which can antagonize the functions of insulin, so that the sensitivity of the pregnant woman's target tissues to insulin is reduced, leading to the occurrence of GDM (14, 15). Therefore, placenta has become an important organ for the study of the pathogenesis of GDM. And placenta-specific miRNAs may contribute to the development of GDM. Here, the trophoblast cell line (HTR8/SVneo) was used to detect the effect of *miR-29b* on cell biological behavior. *MiR-29b* mimic or inhibitor was transfected into trophoblast cells. We did not titer the concentration of miRNA mimic to work within physiological levels. However, we found that 50 nM *miR-29b* mimic significantly enhanced the *miR-29b* level and 50 nM *miR-29b* inhibitor evidently reduced the *miR-29b* level. *MiR-29b* over-expression inhibited cell growth, late apoptosis, migration and invasion, and *miR-29b* knockdown promoted cell migration and invasion. The expression of *miR-29b* was reduced in placenta from patients with GDM, which implying that the proliferation and infiltration ability of trophoblast cells was strengthened. Study on the biological behavior of placental cells shown that the proliferation of trophoblast cells in placenta from the pregnant women with GDM was increased (16). CYP1B1 was highly expressed in placentas from women with GDM and promoted extravillous trophoblast activities, including proliferation, migration, and invasion (17). Thus, it can be speculated that *miR-29b* is involved in the process of GDM through promoting the extravillous trophoblast activities.

Usually, miRNAs execute functions through adjusting the expression of their target genes. We searched *miR-29b* targets online and found that ING2, ING3 and *HIF3A* 3′-UTR had *miR-29b* responsive elements, which was highly conserved domain among different species. Dual-luciferase reporter system analysis showed that *miR-29b* inversely regulated *HIF3A* expression in the trophoblast cell, and the binding site in the 3′-UTR of *HIF3A* was specific for *miR-29b*. Restoring *HIF3A* expression could rescue the inhibitory effects of *miR-29b* over-expression on trophoblast cells. Diminishing *HIF3A* expression could restrict the promotion effect of *miR-29b* knockdown on trophoblast cells. These data further confirm that *HIF3A* was the functional target of *miR-29b*. *HIF3A* promotes colorectal cancer cell growth through non-canonical transcription-independent mechanisms (18). Over-expression of miR-300 inhibited proliferation, invasion and migration of non-small cell lung cancer cells *in vitro*, and tumor growth *in vivo* by downregulating *HIF3A* expression (19). All these facts show that *miR-29b* regulates trophoblast cells activities by reversely modulating *HIF3A* expression.

In this study, we found a confusing phenomenon. Over-expression of *miR-29b* inhibited the luciferase activity in HeLa cells, but no significant change in HEK-293T cells. Knockdown of *miR-29b* promoted the luciferase activity in HEK-293T cells, but no significant change in HeLa cells. The results are quite different between the 2 tested cell lines. We ever considered this question because of the different levels of *miR-29b* expression in two cell lines, so we detected the *miR-29b* expression level in HEK and HeLa cells. However, there was no significant difference in *miR-29b* expression between HEK and HeLa cells. Therefore, we speculated that this difference might be due to the complex regulatory network of miRNA in cells. A miRNA is now thought to regulate the expression of many genes. The gene expression patterns were different between HEK and HeLa cells, which may lead to the different effects on the results of Reporter assays.

In conclusion, this study demonstrates that down-regulation of *miR-29b* in placental tissues are interrelated with GDM. Knockdown of *miR-29b* strengthens the trophoblast cell activities through up-regulating *HIF3A*. This study may provide new insights into the pathogenesis of GDM.

## Materials and Methods

### Patients Samples and Tissue Preparation

GDM was diagnosed by OGTT. All subjects were orally administrated with 75 g glucose regardless of body weight. Blood samples were collected at times 0, 1, and 2 hour (h) and the blood glucose were measured. If fasting blood glucose >5.1 mmol/L, 1 h postprandial blood glucose >10.0 mmol/L, or 2 h postprandial blood glucose >8.5 mmol/L, it can be diagnosed as GDM. Placentas from 204 GDM patients and 202 normal pregnant women, beyond 37 weeks of gestation, were collected from Maternal and Child Health Hospital of Haidian District in Beijing, from March 2012 to May 2013. Basic clinical data of GDM and control population were showed in Table 1. The study was approved by Ethics Committee of Research Institute for Family Planning (2011-08). The informed consent was got from all participants. Placentas were divided into two parts: one part was fixed in 4% paraformaldehyde (PFA) diluted in 0.1 M phosphate-buffered saline (PBS) for immunohistochemical and *in situ* hybridization studies; one part was frozen in the −80°C refrigerator for extracting total RNA and protein.

**Table 1:** Basic characteristics of women with GDM.

**Clinical parameters**	**Control (*****N*** **=** **202)**					**Case (*****N*** **=** **204)**				
	**<25**	**25–30**	**30–35**	**>35**	**Total**	**<25**	**25–30**	**30–35**	**>35**	**Total**
Mean of age (Y)	22.93 ± 1.67	27.82 ± 1.16	31.31 ± 1.05	36.63 ± 1.59	29.68 ± 0.53	23.86 ± 1.26	27.86 ± 1.30	31.52 ± 1.03	36.70 ± 1.12	30.91 ± 0.38
Percentage of each age group	7.43% (15/202)	47.03% (95/202)	33.66 (68/202)	11.88 (24/202)		3.43% (7/204)^**^	32.35% (66/204)^**^	48.04% (98/204)^**^	16.18% (33/204)^**^	
Mean of height (cm)	162.13 ± 4.94	160.80 ± 15.78	161 ± 5.23	160.08 ± 4.26	160.95 ± 11.40	164.29 ± 6.52	161.91 ± 4.94	161 ± 4.45	161.18 ± 5.40	161.65 ± 4.84
Mean of weight (kg)	52.90 ± 6.27	55.96 ± 10.38	54 ± 7.37	55.50 ± 7.66	55.32 ± 8.89	59.43 ± 6.21^*^	57.85 + 8.88	60.95 ± 9.46^***^	61.07 ± 7.82^**^	59.92 ± 9.00^***^
Pre-pregnancy BMI (kg/m^2^)	20.17 ± 1.93	21.12 ± 3.23	21.00 ± 2.51	21.55 ± 3.11	21.07 ± 2.91	21.42 ± 2.10	22.23 ± 3.19^*^	23.44 ± 3.24^***^	23.33 ± 2.37^*^	22.95 ± 3.11^**^
The number of abortions	0.87 ± 0.99	0.65 ± 0.91	0.69 ± 1.05	1.33 ± 1.09	0.77 ± 1.01	0.43 ± 0.79	0.55 ± 0.75	0.86 ± 1.18	1.00 ± 1.17	0.76 ± 1.05
The number of SB	0.00	0.05 ± 0.27	0.13 ± 0.38	0.12 ± 0.34	0.08 ± 0.31	0.00	0.09 ± 0.29	0.16 ± 0.47	0.21 ± 0.55	0.14 ± 0.43
Basic systolic BP (mmHg)	115.00 ± 13.36	115.56 ± 11.01	114.44 ± 10.50	114.25 ± 9.70	114.95 ± 10.82	123.00 ± 12.34	115.65 ± 11.04	118.50 ± 11.05^*^	116.88 ± 12.03	117.47 ± 11.29^*^
Basic diastolic BP (mmHg)	69.27 ± 9.84	70.96 ± 9.70	71.68 ± 8.31	70.00 ± 8.04	70.05 ± 9.06	77.71 ± 11.69	71.83 ± 9.34	73.64 ± 8.96	73.79 ± 7.76	73.22 ± 9.02^*^
0 h OGTT	4.55 ± 0.35	4.57 ± 0.36	4.49 ± 0.36	4.47 ± 0.45	4.53 ± 0.37	5.77 ± 0.38^***^	5.33 ± 0.65^***^	5.36 ± 0.77^***^	5.48 ± 0.72^***^	5.77 ± 5.49^**^
1 h OGTT	6.64 ± 1.46	7.63 ± 1.18	7.57 ± 1.29	7.62 ± 1.15	7.52 ± 1.24	9.77 ± 2.63^**^	10.83 ± 1.36^***^	11.06 ± 1.47^***^	10.90 ± 1.60^***^	10.92 ± 1.49^***^
2 h OGTT	5.97 ± 0.63	6.27 ± 1.06	6.34 ± 0.87	6.24 ± 0.95	6.27 ± 0.96	8.31 ± 0.87^***^	8.58 ± 1.28^***^	9.27 ± 1.37^***^	9.58 ± 2.10^***^	9.07 ± 1.52^***^
Pregnancy days	277.06 ± 7.70	274.56 ± 9.03	2.74 ± 7.30	271.78 ± 6.00	274.37 ± 8.10	276.71 ± 6.58	271.83 ± 9.50	272.15 ± 13.18	26,888 ±10.08	271.68 ± 11.47
Fetal sex (F/M)	9/6	44/51	37/31	17/7	107/95	3/4	27/39	46/52	17/16	93/111
Fetal weight (kg)	3.42 ± 0.48	3.40 ± 0.48	3.38 ± 0.4	3.37 ± 0.27	3.39 ± 0.43	3.29 ± 0.31	3.34 ± 0.68	3.35 ± 0.55	3.39 ± 0.59	3.35 ± 0.59

*Y, year; SB, spontaneous abortion; BP, blood pressure; F, female; M, male*.

* *P < 0.05*,

** *P < 0.01*,

*** *P < 0.001*.

### Plasmid Construction and Transfection

The ING2, ING3, *HIF3A* 3′-UTR, and *HIF3A* 3′-UTR-mutant sequences were amplified by PCR from human genomic DNA using the primers in Table 2. After being double digested with *XhoI* and *Xba*I, the PCR products were cloned into pGL3 control vector (Invitrogen, Carlsbad, CA, USA). The coding region of *HIF3A* sequence was amplified and cloned into pcDNA3.1 vector, designated as pcDNA3.1-*HIF3A*. All the constructs were verified by DNA sequencing. Specific siRNAs for scramble and *HIF3A* were purchased from RiboBio Co., Ltd. (Guangzhou, Guangdong, China).

**Table 2:** Primer Sequence.

**Primers**	**Sequence**	**Size**
ING2-XhoF	CCGCTCGAGCGGCTCAAGGTTGAACAATTT	601 bp
ING2-XbaR	GCTCTAGACAAGGTGAAATGACATGCCTAAA	
ING3-XhoF	CCGCTCGAGGATGCAATTGTGGTGCTCTTT	463 bp
ING3-XbaR	GCTCTAGACAAGCTACAGCCTATTCTACCTATC	
HIF3A-XhoF	CCGCTCGAGCCCTCCAGACATGCACTTAC	521 bp
HIF3A-XbaR	GCTCTAGACCTACCAAGGTGAGGTCTTTATG	
HIF3A-Mu1F	GCTTTCTTTCTACAGATTTTACTACTCTTGGTCT	521 bp
HIF3A-Mu1R	TAGTAAAATCTGTAGAAAGAAAGCTGGGGGGTAA	
HIF3A-Mu2F	GTGGCCTATCCAGATTTTTACTATGGGGG	521 bp
HIF3A-Mu2R	CATAGTAAAAATCTGGATAGGCCACTCTCTGAC	

The *miR-29b* mimic, mimic control, *miR-29b* inhibitor, inhibitor control, scramble siRNA control and *HIF3A* siRNA were synthesized by GenePharma (GenePharma Co., Ltd., Shanghai, China), and transfected into cells by the lipofectamine 2,000 (Invitrogen, Carlsbad, CA, USA) according to the manufacture's instruction.

### *In situ* Hybridization

Placentas from 204 GDM patients and 202 normal pregnant women were separately arranged into two tissue microarrays according to the match between the case group and the control group. Tissue microarrays were cut to 6 μm thickness. The sections were hybridized with digoxigenin (DIG)-labeled LNA-MiR-29b probe (LNA-miR-29b sequence: 5′ –DIG-aAcaCtgAtttCaaatGgtgCta-3′) overnight at 42°C. The probe was replaced by DIG-labeled LNA-scrambled probe (LNA-scrambled sequences: 5′-DIG-caTtaAtgTcGgaCaaCtcAat-3′) was served as negative control. After blocked by blocking buffer containing 5% bovine serum albumin (BSA), the sections were incubated with alkaline phosphatase labeled anti-DIG-antibody (Roche, Mannheim, Germany, 1: 200) overnight at 4°C, and colorated with bromochloroindolyl phos-phate/nitro blue tetrazolium (BCIP/NBT; Promega, Madison, WI, USA). Samples were photoed by Pannoramic scan digital slice scanner (3DHISTECH Ltd., Budapest, Hungary).

### Immunohistochemistry

Tissue microarrays including placentas from 204 GDM patients and 202 normal pregnant women were incubated with rabbit anti-HIF3α polyclonal antibody (GeneTex, USA, 1:500), then incubated with HRP-conjugated goat anti-rabbit IgG (Jackson Immunoresearch Laboratories, West Grove, PA, USA). The sections were developed by diaminobenzidine (DAB; Sigma-Aldrich, St. Louis, MO, USA), and then stained with haematoxylin (Sigma-Aldrich, St. Louis, MO, USA). Normal goat serum instead of primary antibody was served as negative control. Samples were observed by Pannoramic scan digital slice scanner (3DHISTECH Ltd., Budapest, Hungary). The MOD of positive signals was determined through Image J software with H-score grading system in three different optical fields selected in a random manner for each sample. Random 15 pair samples in each age group (≤ 25 Y, 26–30 Y, 31–35 Y, ≥36 Y) were selected for optical density analysis.

### qRT-PCR to Quantify *miR-29b* Expression

Total RNAs from human placentas were isolated using Trizol reagent (Invitrogen, Carlsbad, CA, USA). The expression of *miR-29b* was detected by qRT-PCR using TaqMan MicroRNA Reverse Transcription Kit and TaqMan Univel PCR Master Mix (Applied Biosystems, Foster City, CA, USA) according to the manufacturer's instructions. U6 snoRNA was used for normalization. Random 10 pair samples in each age group (OGTT 1st h, 2nd h, 0 and 1st h, 0 and 2nd h, 1st and 2nd h, 0 and 1st and 2nd h) and random 15 pair samples in each age group (≤ 25 Y, 26–30 Y, 31–35 Y, ≥36 Y) were selected for qRT-PCR. Each sample in each experiment was detected in triplicate.

### MTT Assay

The effects of *miR-29b* on human trophoblasts cells viability were estimated with MTT assay. HTR8/SVneo cells were seeded in 96-well plate (5,000 cells per well) and allowed to attach overnight. Cells were transfected with 50 nM *miR-29b* mimic, miRNA mimic negative control, *miR-29b* inhibitor or miRNA inhibitor negative control, respectively. After 48 h, 20 μL MTT (5 mg/mL; Promega, Madison, WI, USA) was added to each well and incubated for 4 h at 37°C. The supernatant was then removed and the 150 μL dimethyl sulfoxide (Sigma-Aldrich, St. Louis, MO, USA) was added into each well. The absorbance value was recorded at A570 nm with a 96-well plate reader (Bio-Rad 3550; Bio-Rad, Hercules, CA, USA). Each treatment group had three replicates and the experiment was repeated for three times (*n* = 9).

### EdU Assay

EdU is a thymidine analog which can infiltrate into the replicated DNA molecule instead of thymidine during cell proliferation to label proliferative cells. The effects of *miR-29b* on human trophoblasts cells proliferation were analyzed with Cell-Light^TM^ EdU Apollo®567 *in vitro* Imaging Kit (RiboBio, Guangzhou, Guangdong, China) according to the manufacturer's instructions. Briefly, HTR8/SVneo cells were exposed to 50 μM EdU for 2 h at 37°C after 48 h transfection of 50 nM *miR-29b* mimic, miRNA mimic negative control, *miR-29b* inhibitor or miRNA inhibitor negative control, respectively. Then cell nuclei were stained with Hoechst 33342. The samples were viewed and imaged under a laser-scanning confocal microscope (CarlZeiss LSM 710 META, Germany). Proliferative cells and total cell number were separately computed in three fields (magnification × 400) selected in a random manner. Each treatment group had three replicates and the experiment was repeated for three times (*n* = 9). The results were represented as a ratio of the number of proliferative cells vs. total cells.

### Flow Cytometry Analysis

The effects of *miR-29b* on human trophoblasts cells apoptosis were analyzed with by annexin V-FITC staining kit (Invitrogen, Carlsbad, CA USA) according to the manufacturer's instructions. Briefly, HTR8/SVneo cells were harvested after 48 h transfection of 50 nM *miR-29b* mimic, miRNA mimic negative control, *miR-29b* inhibitor or miRNA inhibitor negative control, respectively. Each sample added 5 μL Alexa Fluor® 488 annexin V and 1 μL PI. After 15 min of incubation, each sample added 400 μL 1 × annexin V-binding buffer. The samples were detected at a rate of 8,000 events per second by flow cytometry (BD Biosciences, San Jose CA, USA). The experiment was repeated for three times (*n* = 3).

### Cell Migration and Invasion Assay

Cell migration and invasion were assayed using a Transwell chamber (Corning, NY, USA). HTR8/SVneo cells transfected with 50 nM *miR-29b* mimic, miRNA mimic negative control, *miR-29b* inhibitor or miRNA inhibitor negative control were seeded on the top chamber of 24-well plate with or without 40 μL of 1 mg/ml Matrigel (BD, Franklin Lakes, NJ, USA). After 18 h of incubation for migration assay or 24 h for invasion assay, the non-migrated or infiltrated cells on the top of chamber were carefully removed with a cotton swab, and the migrant or invasive cells on bottom of chamber were fixed with 4% formaldehyde. The cells were then stained by hematoxylin and eosin (Sigma-Aldrich, St. Louis, MO, USA). The cells were viewed and imaged under a microscope (BX61; Olympus, Tokyo, Japan). Three different fields (magnification × 400) were selected in a random manner to calculate the number of migration or invasion cells. The results were represented as the average number of cells per field. The experiment was repeated for three times (*n* = 3).

### Western Blot Analysis

Extracted protein was boiled in SDS/β-mercaptoethanol sample buffer. Sixty microgram protein was run on 6–10% gradient polyacrylamide gel and transferred to PVDF membranes (Amersham, St Albans, Herts, UK). The membranes were incubated with rabbit anti-HIF3α polyclonal antibody (GeneTex, USA, 1:1000) or mouse anti-β-ACTIN monoclonal antibody (Abcam, Cambridge, MA USA, 1:1000) 2 h at 27°C. Then the membranes were incubated with horseradish peroxidase (HRP)-conjugated goat anti-rabbit IgG or goat anti-mouse IgG (Jackson Immunoresearch Laboratories, Inc., West Grove, PA, USA, 1:10,000) 1 h at 37°C. ECL The membranes were incubated with ECL reagents 1 min (Millipore, Billerica, MA, USA) and exposed to X-ray film (Kodak, USA). The β-ACTIN was used as a loading control. The experiment was repeated at least three times. The bands were analyzed using Quantity One analyzing system (Bio-Rad, Hercules, CA, USA). The protein level was represented as the relative ratio of the HIF3α signals vs. β-ACTIN signals. The detection of HIF3α protein level in HTR8/SVneo cell transfected with *miR-29b* mimic and inhibitor was repeated for three times (*n* = 3).

### Dual-Luciferase Activity Assay

Partial sequence of the 3′-UTR from *HIF3A* and mutating *miR-29b* target site in the 3′-UTR of *HIF3A* were cloned into the downstream of the firefly luciferase gene in PmirGLO Vector, respectively (Promega, Madison, WI, USA) using primers in Table 2. HEK-293T cells or HeLa cells were seeded in 48-well plates and attached overnight, then transfected using lipofectamine 2,000 (Invitrogen, Carlsbad, CA, USA). 48 h later, cells were detected by the dual-luciferase assay (Promega, Madison, WI, USA). The detection of binding ability of *miR-29b* and *HIF3A* 3′-UTR was performed three separate experiments. Each treatment group was repeated for *three* times for independent triplicate experiments (*n* = 9). The results were expressed as relative luciferase activity (Firefly LUC/Renilla LUC).

### Statistical Analyses

Data are presented as means ± standard deviations (SD) and the error bars represent SD. Comparisons between two groups (paired and unpaired) were performed using a two-tailed Student's *t*-test. One-way analysis of variance (ANOVA) and Turkey's *post-hoc* test was used to compare data sets with more than two groups. *P* < 0.05 was considered statistically significant. All of the statistical analyses were performed with SPSS 16.0 software.

## Data Availability Statement

The raw data supporting the conclusions of this article will be made available by the authors, without undue reservation, to any qualified researcher.

## Ethics Statement

The studies involving human participants were reviewed and approved by Ethics Committee of Research Institute for Family Planning. The patients/participants provided their written informed consent to participate in this study.

## Author Contributions

D-GS performed the experiments and drafted the manuscript. ST collected clinical tissues and patients information. YH was responsible for qRT-PCR. LZ and C-YG was responsible for samples collecting and recording. XM was responsible for institute management. H-FX designed the research and revised the manuscript.

### Conflict of Interest

The authors declare that the research was conducted in the absence of any commercial or financial relationships that could be construed as a potential conflict of interest.
