# Supplementary material for: The miRNA-29b Is Downregulated in Placenta During Gestational Diabetes Mellitus and May Alter Placenta Development by Regulating Trophoblast Migration and Invasion Through a HIF3A-Dependent Mechanism
Source: Front Endocrinol (Lausanne). 2020 Mar 31;11:169. doi: 10.3389/fendo.2020.00169 (PMC7137738; doi:10.3389/fendo.2020.00169)
Supplement: Supplementary file 1 [file Table_1.DOC]

**Supplemental data**

**
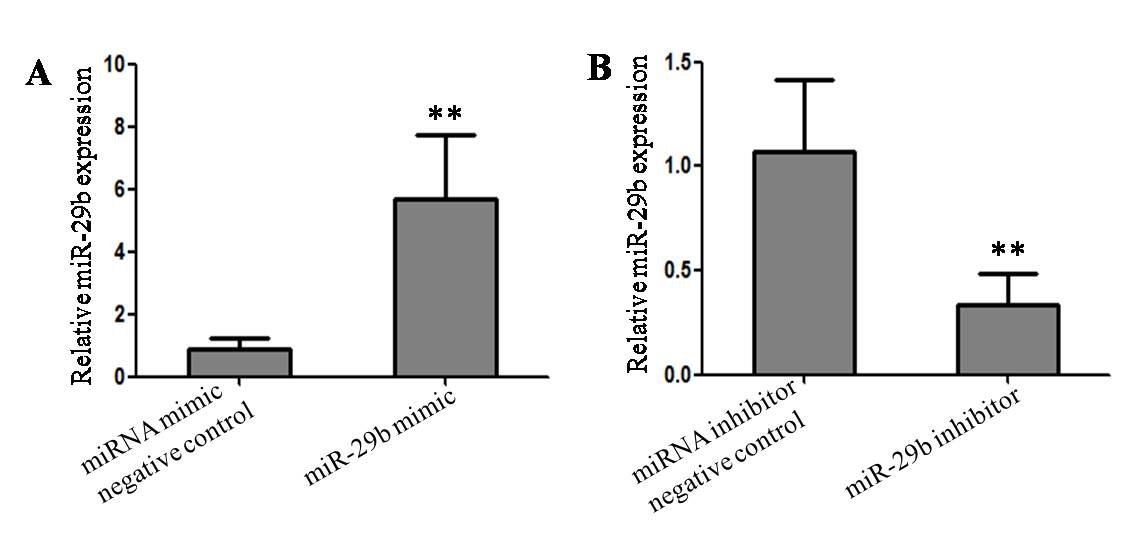
**

**Figure S1 *miR-29b* expression detection in HTR-8/SVneo cells after transfected with *miR-29b* mimic or *miR-29b* inhibitor.** *MiR-29b* expression level was detected in HTR-8/SVneo cells transfected with *miR-29b* mimic and miRNA mimic negative control (A), or *miR-29b* inhibitor and miRNA inhibitor negative control (B).

**
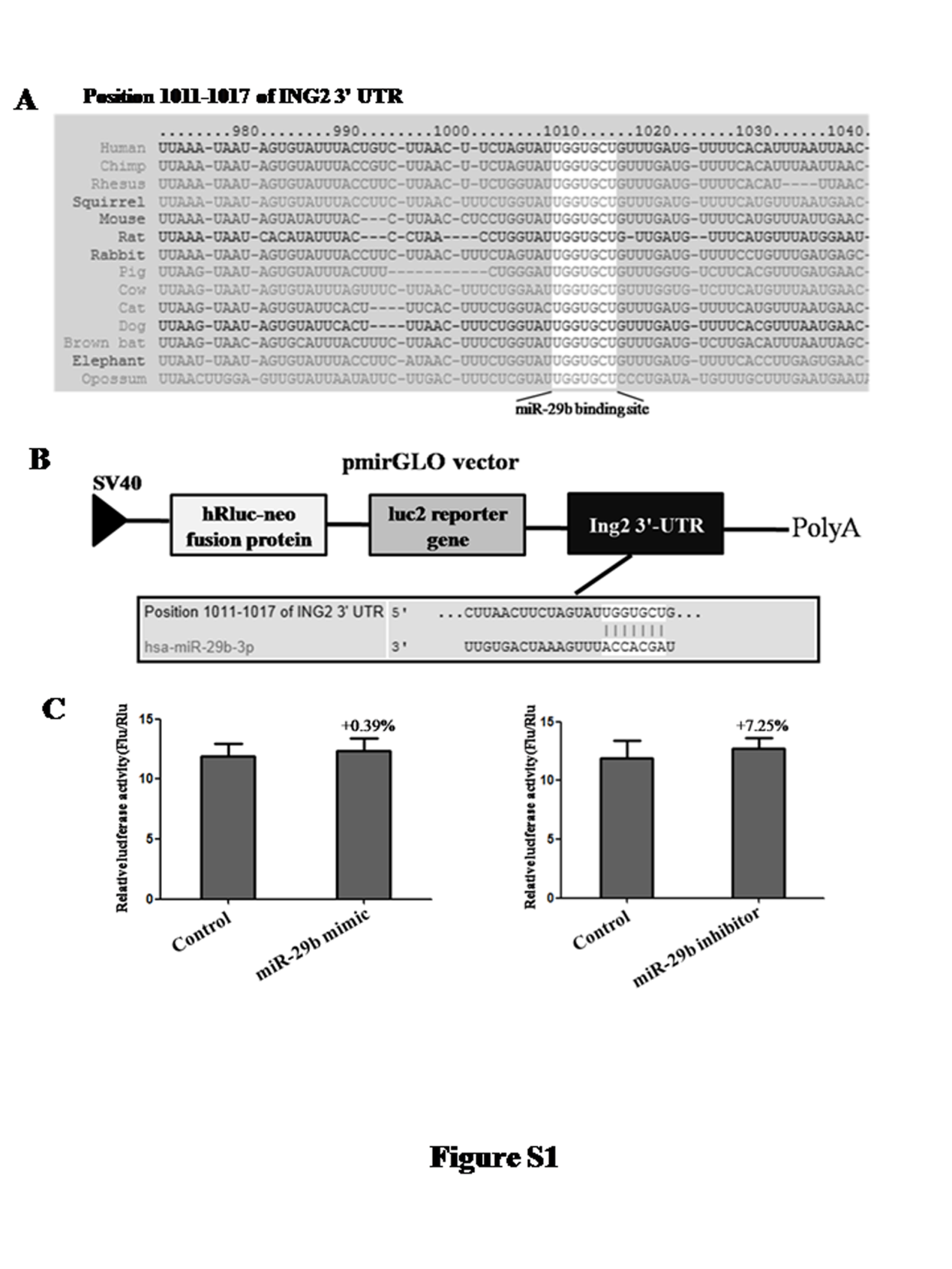
**

**Figure S2 The prediction and confirmation of the *miR-101* target ING2.** (A) *MiR-29b* binding sites in the 3'-UTR region of ING2 in cross-species. **(**B) Schematic diagram for constructing the *miR-29b* binding site into pmirGLO vector. (C) Confirmation of the target gene of *miR-29b*. HEK-293T cell cells were co-transfected with miRNA mimic control, *miR-29b* mimic, miRNA inhibitor control or *miR-29b* inhibitor and HIF3α-pmirGLO for dual-luciferase assay.

**
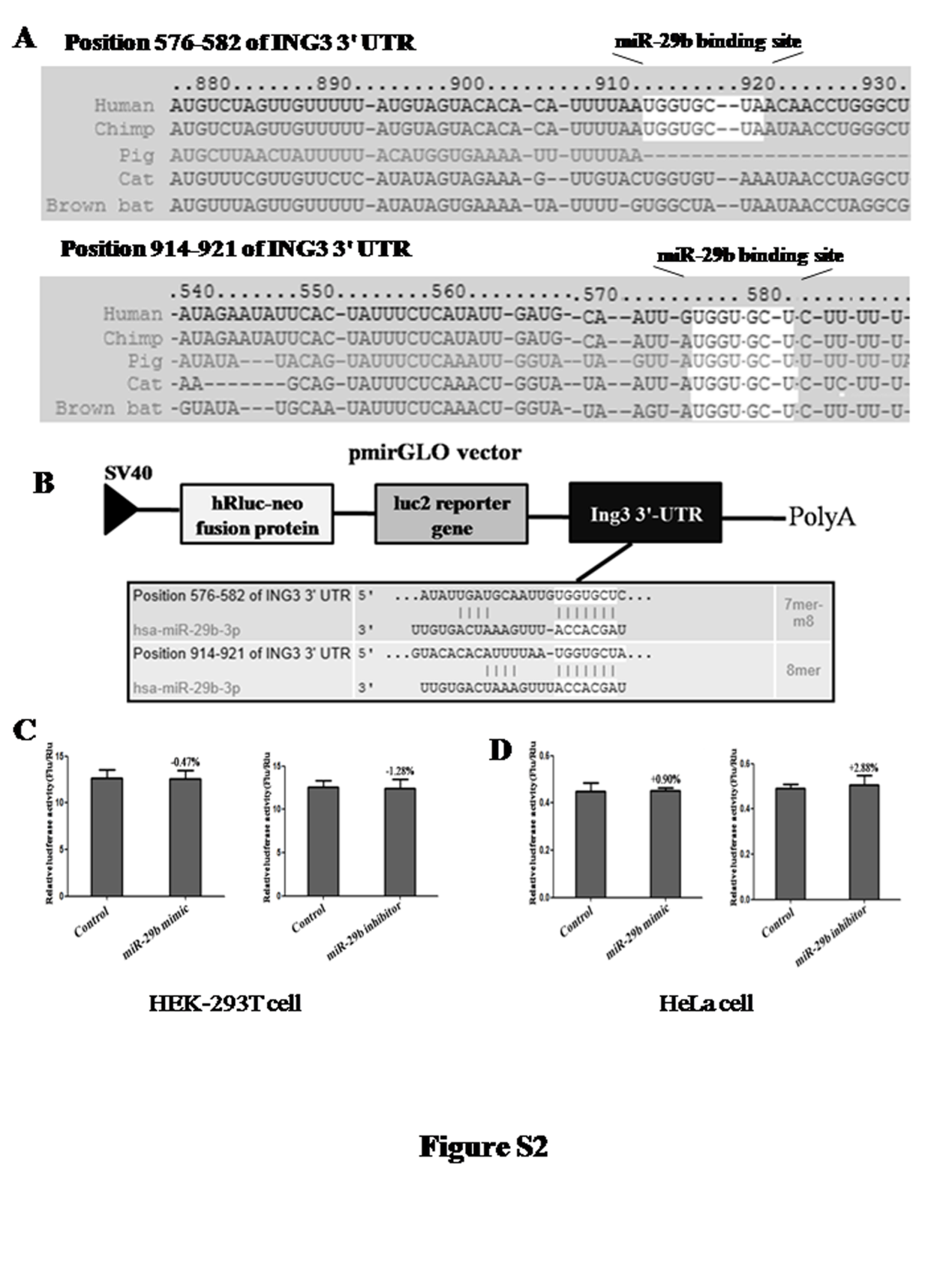
**

**Figure S3 The prediction and confirmation of the *miR-101* target ING3.** (A) *MiR-29b* binding sites in the 3'-UTR region of ING3 in cross-species. **(**B) Schematic diagram for constructing the *miR-29b* binding site into pmirGLO vector. (C) Confirmation of the target gene of *miR-29b*. For dual-luciferase assay, HEK-293T cells were co-transfected with miRNA mimic control, *miR-29b* mimic and HIF3α-pmirGLO; HeLa cells were co-transfected with miRNA inhibitor control or *miR-29b* inhibitor and HIF3α-pmirGLO.
